# The psychosocial challenges experienced by students with disabilities at the University of Technology in South Africa, KwaZulu-Natal

**DOI:** 10.4102/ajod.v14i0.1668

**Published:** 2025-10-08

**Authors:** Andile S. Masuku, Maureen N. Sibiya, Dumile Gumede, Reggiswindis T. Hlengwa

**Affiliations:** 1 Student Governance and Development, Student Services, Durban University of Technology, Durban, South Africa; 2 Office of the Vice-Chancellor, Mangosuthu University of Technology, Durban, South Africa; 3 Executive Deans Office, Faculty of Health Sciences, Durban University of Technology, Durban, South Africa; 4 Department of Community Health Studies, Faculty of Health Sciences, Durban University of Technology, Durban, South Africa

**Keywords:** University of Technology, disabilities, psychosocial, student affairs, practitioners, KwaZulu-Natal

## Abstract

**Background:**

Students with disabilities encounter numerous challenges in life, which ultimately harm their psychosocial well-being. These psychosocial challenges pose a threat to their university life experience and hinder the chances of success for students with disabilities.

**Objectives:**

The study aims to explore the perspectives of student affairs practitioners and students with disabilities on the psychosocial challenges of students with disabilities.

**Method:**

In this study, we used data from semi-structured interviews with 12 purposively selected student affairs practitioners and students with disabilities. After transcription, the data were analysed thematically.

**Results:**

Four themes were identified: (1) poor infrastructure and a lack of resources; (2) stigmatisation and discrimination; (3) mental health and social challenges; and (4) awareness of disability and provision of adequate resources.

**Conclusion:**

Students with disabilities face several psychosocial challenges and these challenges harm their mental health and social well-being.

**Contribution:**

This study adds to the expanding body of knowledge on the psychosocial challenges of students with disabilities. Universities have committed to advocating for and ensuring the inclusivity of these students. Consequently, it is crucial to understand their daily challenges and the necessary interventions to address them, ensuring that these students feel a sense of belonging and recognition.

## Introduction

Institutions of higher education formally afford students opportunities to develop their talents and skills; for this to be achieved, students must have access to relevant resources. However, this has not been the case for students with disabilities as they are confronted with numerous challenges (Nene 2019). People with disabilities include those who have long-term physical, mental, intellectual or sensory impairments, which may prevent their full and effective participation in society (Ginis et al. 2021). Accessing university-level education can be met with many hindrances, especially for students with disabilities (Madhesh 2023). Students with disabilities may face challenges when reaching higher education and attempting to be successful (Martins, Melo & Martins 2021). People with disabilities experience challenges in higher education settings with the inclusive educational principles of the institutions (Moriña, López-Gavira & Morgado 2017). Inclusive education in universities is still inadequate for numerous reasons, including low expectations, negative attitudes, inadequate or irrelevant teacher training, physical barriers, availability of resources, transitional planning and ineffective support services (Sánchez, De Haro-Rodríguez & Martínez 2019). The lack of inclusive educational practices constitutes significant academic barriers for students with disabilities (Algolaylat et al. 2019).

Inclusion is about providing equitable opportunities to all students, including those with challenges, to receive services with the necessary supplemental aids and support that conform to their needs (Hill, Shaewitz & Queener 2020). Disability and all its related aspects pose significant challenges for individuals, families and communities worldwide (Makwela & Smit 2022). It is a known fact that disability in any form or shape is a phenomenon that incurs social, emotional and learning problems (Moasun 2023). Hence, students with disabilities face several psychosocial challenges that could vary from home to home depending on family backgrounds, and the educational and economic statuses of the caregivers (Abeshu & Baissa 2018). Therefore, this study is important as it aims to explore the perspectives of student affairs practitioners and students with disabilities on psychosocial challenges. The motivation for this study comes from Mutanga (2018), who highlighted that globally, few students with disabilities progress in higher education, with a particular focus on the psychosocial challenges faced in the South African context because of its complex history and socio-economic issues. These barriers affect students from the start of their undergraduate studies through to completion. Even those who manage to access higher education still face significant challenges. Therefore, it is crucial to explore the psychosocial challenges of students with disabilities through the experiences of both student affairs practitioners and the students themselves. Identifying intervention and prevention strategies to address these challenges is essential.

### The psychosocial challenges of students with disabilities in higher education

The right to a decent, high quality and acceptable life for people with disabilities has been the most important principle of many international laws and conventions over the past years (Madhesh 2023). These international laws include the United Nations Convention on the Rights of Persons with Disabilities (2006), which advocates for equal rights, accessibility and social inclusion for individuals with disabilities (Watson et al. 2022). Another relevant international framework is the Sustainable Development Goals (SDGs) (2015), as the 2030 Agenda for Sustainable Development outlines specific objectives for the inclusion of persons with disabilities in education, employment and social protection (Hajian & Kashani 2021). In addition, Guematcha (2022) highlights the African Charter on Human and Peoples’ Rights (ACHPR) and the Protocol on the Rights of Persons with Disabilities (2018), which address disability rights within the African context, emphasising inclusion and non-discrimination.

The number of students with disabilities, entering higher education institutions, has increased (Mantsha 2016). Inclusive disability legislation played a crucial role in increasing the number of students entering higher education; however, barriers to the full participation of students remain (Biggeri, Di Masi & Bellacicco 2020). García-González et al. (2021) argue that students with disabilities are exposed to barriers that relate to the lack of guidance and information at the time of entering university. Thus, it is vital to introduce measures that would facilitate this process mainly to modify policies for improving attention to disability. Even though universal inclusion is a basic principle of SDGs, the inclusion of people with disabilities in humanitarian interventions and policies is still intangible (Trani et al. 2020). Moreover, in the last two decades, the South African government has developed impressive policies for the inclusivity and accommodation of students with disabilities in higher education (Lourens & Swartz 2020). However, a concern was raised regarding the South African higher education system that it has inadequate resources and the policies are slowly and poorly executed (Carrim & Wangenge-Ouma 2012). Mpu and Adu (2021) explain that the Inclusive Education Policy (White Paper 6 2001) aims to integrate learners with disabilities into mainstream education while providing special schools where necessary. However, its implementation has been inadequate because of a lack of resources, inaccessible infrastructure and untrained teachers, preventing many children with disabilities from receiving a proper education. In South Africa, several comprehensive policies are in place, concerning education and the rights of people with disabilities. Nonetheless, the execution of these policies is considered weak (Moodley 2021). It must be noticed that despite the challenges, related to the implementation of policies, the South African government is making efforts to ensure that people with disabilities have enhanced access to quality education and employment (Zongozzi 2022).

The stigma and its effects on the life course trajectories of people with disabilities have raised interest, but, sadly, students with disabilities are not exempted from this matter (Chatzitheochari & Butler-Rees 2023). Moreover, exposure to stigma and stereotypical threats is damaging for several marginalised groups (Haft, Greiner de Magalhães & Hoeft 2023). People with disabilities have been frequently stigmatised and it has harmful impacts concerning collective socio-educational inclusion (Gallego-Ortega & Rodríguez-Fuentes 2021). Majoko (2018) concurs that students with disabilities have historically been marginalised and discriminated against in higher education. People with disabilities are vulnerable to discrimination, which leads to isolating themselves from society because they feel unwanted and rejected. This harms their psychosocial well-being (Hazarika 2019). Disability can be a traumatic experience for others as it usually comes with long-term effects, which may contribute to psychological challenges, related to anxiety, depression and poor psychological wellness (Oyeleke & Busari-Akinbode 2021). However, a growing perspective among people with disabilities has shifted the conceptualisation of disability as a state of weakness and disadvantage (Onalu et al. 2024). Furthermore, disability may also reduce one’s internal coping resources, such as mastery and self-esteem, external coping resources and social support (Namkung & Carr 2020).

Students with disabilities are confronted with the same challenges as other university students, such as academic progress, social connections, finances and other unexpected challenges. However, students with disabilities are often in need of extra assistance regarding their physical, mental and developmental differences (Hlengwa & Masuku 2022; Safer, Farmer & Song 2020). Despite the various challenges of students with disabilities, efforts have been made to create an educational culture where students feel competent, valued and not excluded. Irrespective of their characteristics, interests, abilities or difficulties, access for people with disabilities has become a legally recognised right (Fernández-Batanero, Montenegro-Rueda & Fernández-Cerero 2022). Support services and legislation have greatly contributed to the steady increase of students with disabilities in higher education (Yssel, Pak & Beilke 2016). Hence, globally, universities have initiated a gradual transformation in responding to student diversity. During this process, universities have started analysing the needs of students with disabilities to improve the accessibility of all their services and resources (Carballo et al. 2023). However, despite these efforts of the universities, the literature shows that much must still be done to achieve different perspectives (Moriña, Sandoval & Carnerero 2020). It is therefore important that higher education institutions should consider the voices of vulnerable groups of students to reduce existing barriers effectively (Rodríguez Herrero, Izuzquiza Gasset & Cabrera Garcia 2020). Inasmuch as progress has been made in providing access to students with disabilities, more awareness of disability, anti-stigma and anti-discrimination training are still needed (Kauffman et al. 2022). It is also recommended that the training and awareness of the whole university community must be improved, particularly, on matters, related to students with disabilities (García-González et al. 2021; Hlengwa & Masuku 2022).

### Theoretical framework

The study was guided by -the socio-ecological framework. Bronfenbrenner developed the socio-ecological model originally to understand factors that influence human development better (Tebb & Brindis 2022). The socio-ecological framework is a multilevel conceptualisation of health that involves intrapersonal, interpersonal, organisational, environmental and public policy factors (Scarneo et al. 2019). Unlike linear or single-factor frameworks, the socio-ecological framework emphasises that people are embedded in different systems and that the interaction that is happening between these systems impacts their health and well-being (Bueno-Gutierrez & Chantry 2015). The socio-ecological framework was applied to this study to analyse the various levels influencing students with disabilities at the University of Technology (Hlengwa & Masuku 2022). It examines challenges from multiple perspectives: the individual level focuses on personal experiences; the interpersonal level looks at relationships with family, peers and teachers; the institutional level explores educational structures and policies; the community level considers the broader environment; and the societal level addresses wider cultural and societal influences. By using this framework, the study moved beyond individual challenges, placing students’ experiences within a broader institutional and societal context.

## Research methods and design

### Research design

This study was guided by a qualitative narrative research design as it seeks to explore the perspectives of student affairs practitioners and students with disabilities on psychosocial challenges that students with disabilities experience. This method allows the researcher to gather and examine in-depth personal narratives to gain insight into individuals’ lived experiences, perspectives and the meanings they attribute to significant life events (Hlengwa & Masuku 2022; Renjith et al. 2021).

### Study setting

This study was conducted at the University of Technology in KwaZulu-Natal (KZN). This is a public university where student affairs professionals from different units work under the Student Services Units.

### Sampling and participants

The study used a purposive sampling technique to select participants who aligned with the study’s objectives. The criteria for selection included student development officers, psychologists, professional nurses and disability officers, as these student affairs practitioners interact regularly with students with disabilities and are knowledgeable about their challenges. Participants were required to be over 18 years old, with student affairs practitioners having at least one year of experience. Five students with disabilities and seven student affairs practitioners were chosen. Staff members not involved in student affairs and students without disabilities were excluded. This sample size aligns with qualitative research standards for meaningful insights, similar to previous studies on psychosocial challenges and disability support in higher education. Table 1 illustrates the demographic characteristics of participants.

**TABLE 1:** Demographic characteristics of participants.

Participant number	Gender	Race	Age range (years)	Representation
P1	Male	Black person	26–30	Student
P2	Female	Black person	18–25	Student
P3	Male	Black person	26–30	Student
P4	Male	Black person	18–25	Student
P5	Female	Black person	18–25	Student
P1	Female	Black person	31–35	Practitioner
P2	Female	Black person	41–45	Practitioner
P3	Male	Black person	31–35	Practitioner
P4	Female	White person	41–45	Practitioner
P5	Female	Black person	46–50	Practitioner
P6	Male	Black person	26–30	Practitioner
P7	Female	Black person	51–55	Practitioner

### Recruitment process

The researchers obtained permission from the University’s Gate Permission Committee after receiving provisional ethics clearance from the Institutional Research Ethics Committee (IREC). Researchers then approached student affairs unit managers and the Disability Rights Unit through the Student Counselling and Health Unit to recruit participants. An information letter outlining the study’s aim and procedures was provided, and participants were asked to sign a consent form. Those who did not consent were excluded from the study.

### Data collection

The interviews were semi-structured to provide flexibility in data collection. This approach allowed the researcher to probe deeper, encouraging participants to elaborate or explore new topics based on their responses. Therefore, participants have the freedom to express their views in their own words (Adeoye-Olatunde & Olenik 2021; Hlengwa & Masuku 2022). The interview guide was designed to align with the study’s objectives. Face-to-face interviews, lasting 30 min to 40 min, were conducted with 12 participants, including student affairs practitioners and students with disabilities. The interviews were audio-recorded for data analysis, and data collection continued until saturation was reached.

### Data analysis

The researchers transcribed the recorded interviews verbatim. For data analysis purposes, researchers used Tesch’s eight steps in the coding process (Creswell 2014). The researchers carefully reviewed the transcripts multiple times to enhance their understanding of the perspectives of both student affairs practitioners and students with disabilities. They avoided abbreviating categories, instead grouping similar topics into themes and sub-themes, which were then coded and categorised. Field notes were also used to provide context during analysis. After a preliminary analysis and discussion, the researchers reached a consensus on the final themes. By applying the socio-ecological framework, they identified interconnected influences on students’ experiences, leading to a deeper understanding and informed recommendations for addressing the challenges faced.

### Enhancing trustworthiness

To ensure the trustworthiness of the study, as Ahmed (2024) describe, four criteria of credibility, dependability, conformability and transferability were used. Credibility was measured by ensuring that the research findings accurately reflect the experiences and perspectives of the participants. Dependability was achieved by internally conducting an inquiry audit to demonstrate the consistency and reliability of the research findings. Confirmability was achieved by ensuring the collected data were checked throughout data collection and analysis. The researchers ensure validity, reliability and ethical research practices to minimise bias and enhance objectivity. Transferability was achieved through the thick description of the research findings from the collected data. This eventually involved providing adequate details on the study setting, participants and methods that were used to collect the data.

### Ethical considerations

The study received ethical clearance from Institutional Research Ethics Committee on 03 July 2024 (No. IREC 031/24) and obtained written consent from participants. An informational letter outlined the research procedure, emphasising voluntary participation with the option to withdraw at any time. Interviews were audio-recorded with permission, and pseudonyms were used to protect participants’ identities. Participants were informed about the availability of psychological support, although no requests for such support were made. Data were securely stored on a password-protected computer, with audio recordings kept on an external hard drive. After five years, all electronic data will be deleted and hard copies shredded (Hlengwa & Masuku 2022).

## Results

Table 2 outlines the themes that emerged from the study.

**TABLE 2:** Themes that emerged from the study.

Number	Theme
Theme 1	Poor infrastructure and a lack of resources
Theme 2	Stigmatisation and discrimination
Theme 3	Mental health and social challenges
Theme 4	Awareness of disability and provision of adequate resources

### Theme 1: Poor infrastructure and a lack of resources

Students with disabilities face major challenges on campus because of poor infrastructure and limited resources, which hinder their learning and mobility. While some student affairs practitioners acknowledge the university’s support, students feel that resources are insufficient and often experience marginalisation despite efforts to accommodate them. The following excerpts illustrate this experience:

‘… [*S*]ometimes we cannot even attend other lectures on campus because of venues that are not conducive for our learning. I remember this other time, we were told that the lecture would be on this particular campus, and when I got there, there was no lecture only to find that it had been moved to another campus because of issues with venue bookings. I ended up not attending that lecture because I could not go to that campus.’ (P5, female, student, 18–25 years old)

‘For me, I think we do not have good infrastructure and resources that accommodate the needs of students with disabilities. Yes, we are trying by all means to ensure that they feel accommodated especially in our residences, but on campus, we are still struggling to provide them with necessary support.’ (P3, male, student affairs practitioner, 31–35 years old)

‘…. [*W*]e try by all means to ensure that students with disabilities have support from the university, but the problem is that there is a lack of resources.’ (P7, female, practitioner, 51–55 years old)

‘… [*I*]n my own experience, we do not receive enough support with the resources that we need for our special needs. Even when we attend programmes, there is a slim chance that we will find interpreters for deaf students. Sometimes, the venues where they host these programmes do not accommodate students with disabilities. If you need something from campus, you hardly get assistance because there are no resources for us.’ (P1, male, student, 26–30 years old)

Participants’ experiences highlight the significant barriers that students with disabilities face in accessing and participating fully in university life. Inaccessible lecture venues and difficulties moving between campuses often disrupt their learning and academic progress. While some efforts have been made by the university to provide support, these initiatives are constrained by resource limitations, leaving students feeling marginalised rather than accommodated.

### Theme 2: Stigmatisation and discrimination

The findings of the study indicate that most students with disabilities feel their peers stigmatise and discriminate against them. Many people believe their disabilities make them different, which often results in stigmatisation (Hlengwa & Masuku 2022). Moreover, the study revealed concerns about discrimination; students with disabilities felt they were treated unfairly because of their conditions and often overlooked because others assumed they lacked capability. This experience contributes to a lack of confidence and leads to feelings that their emotions and needs are not valued. Participants said:

‘… [*B*]ecause of our disabilities, they think that we are different from them and that is why they stigmatise us and label us. I have been in a situation where I was stigmatised by my residence mates.’ (P4, male, student, 18–25 years old)

‘Students with disabilities also experience discrimination and I have dealt with a case where the student with disability expressed her concern about the situation she was faced with where she was discriminated by her classmates. This is wrong and it kills their self-confidence because they are being overlooked because of their disabilities.’ (P4, female, practitioner, 41–45 years old)

‘… [*T*]here are several psychosocial challenges that are faced by the student with disabilities which include being stigmatised by people who do not understand disability and who are not considerate of their feelings because we know that in general, people with disabilities are vulnerable.’ (P6, male, practitioner, 26–30 years old)

Participants shared that they feel stigmatised by their peers, who often view them as different, leading to labelling and unfair treatment. Discrimination exacerbates these challenges, causing students with disabilities to feel overlooked and undervalued, which undermines their confidence and sense of belonging. Some participants found that students with disabilities are frequently isolated and neglected, particularly in extracurricular activities, leading to feelings of loneliness as they are often excluded and treated as an afterthought. This is what the participants said:

‘… [*W*]hen there are student programmes, it is difficult for some of us to attend because we know our needs will not be catered for. There was this time in my residence when we had a programme on the fourth floor and there are no lifts, you have to use the stairs to get to that floor. As much as I wanted to attend the programme, I could not go because of my condition. This tells you that we are isolated, maybe not intentionally, but I felt like we are not taken into consideration.’ (P3, male, student, 26–30 years old)

‘… [*M*]ost of the time, I would feel lonely because I know that I cannot do the things I like and be with people I would want to be with because I feel like a burden and the university sometimes acts like we are an afterthought because they do not give us enough support.’ (P5, female, student, 18–25 years old)

The exclusion of students with disabilities from extracurricular activities and social events, whether intentional or not, fosters isolation and loneliness.

### Theme 3: Mental health and social challenges

The study identified mental health and social challenges as the most significant issues faced by students with disabilities, severely affecting their overall well-being. Poverty also emerged as a major concern, with many students lacking the resources needed to meet their basic needs. The lack of peer support exacerbates mental health struggles, as students often find it hard to connect with others. In addition, difficulties in forming relationships are often linked to low self-esteem. Participants emphasised the following points as critical factors, affecting their experiences:

‘…. [*P*]overty is also a challenge that is faced by students with disabilities. Some of the students come from homes which are economically challenged where they cannot meet the special needs of these students.’ (P2, female, practitioner, 41–45 years old)

‘… I had experienced psychological issues because of my disability. At the time it was difficult for me to accept my physical condition and the lack of support from my peers also played a role in my mental health breakdown because I was not able to communicate my feelings with others.’ (P2, female, student, 18–25 years old)

‘… [*F*]or me is the inability to create lasting relationships with others, in as much as I try to engage with people but because of my confidence and poor communication skills, I cannot relate with my peers. The other issue is that disability also affects my physical health as there are times when I cannot move because of back pain and the day would pass without me doing anything, especially my academic work.’ (P1, male, student, 26–30 years old)

Many participants noticed that students with disabilities face psychological challenges, including difficulty accepting their disabilities and a lack of emotional support from peers, which worsens feelings of isolation and negatively affects their mental health. Social issues, such as struggles with forming meaningful relationships, low self-confidence and exclusion from university programmes, further intensify these challenges.

The psychosocial challenges faced by students with disabilities also hinder their academic progress, including participation in extracurricular activities. It was emphasised that the primary reason for students to be at university is their academic goals, and they must receive the necessary support to achieve academic success and excellence. However, the views from participants stated otherwise:

‘… Instead of focusing on their academic work, students with disabilities often have to redirect their energy towards overcoming the barriers they face. Many times, they miss lectures because of challenges that impact their well-being, preventing them from addressing the most important tasks. I always encourage my colleagues to implement proper measures that break down these barriers and support the academic success of students with disabilities.’ (P5, female, practitioner, 46–50 years old)

‘… [*Y*]ou would notice that in most of the programmes that are hosted by the university, we do not participate and some of the programmes are important for us to attend but we are excluded because our needs are not accommodated.’ (P1, male, student, 26–30 years old)

‘… It is rare for students with disabilities to be recognised for academic excellence because of the challenges they face in completing their academic work, largely caused by teaching and learning barriers. Unfortunately, these challenges significantly impact their ability to focus on their primary goal – academic success. It is crucial to ensure that they receive the necessary support to overcome these obstacles and succeed in their academic pursuits.’ (P1, female, practitioner, 31–35 years old)

Economic hardships, especially for students from disadvantaged backgrounds, worsen these challenges by restricting access to essential resources. Physical health issues also hinder their ability to fully participate in academic and social activities, often resulting in missed lectures and reduced academic performance.

### Theme 4: Awareness of disability and provision of adequate resources

Participants highlighted intervention and prevention strategies to address the psychosocial challenges faced by students with disabilities. The study suggests that the university should invest more in raising awareness about disabilities to educate the community. In addition, providing adequate resources, especially in residences, is crucial to improving the living experiences of these students. The following quotes reflect the participants’ perspectives:

‘… [*T*]he university needs to invest more in creating awareness on disability because I feel like other people are not educated on disability and the types of disabilities that exist. This awareness is important most especially to those who are dealing with students with disabilities so that they are equipped with skills and knowledge on disability.’ (P5, female, student, 18–25 years old)

‘… [*F*]or now, we do not have enough resources to assist students with disabilities, especially in our residences. Our residences are good but they do not have spaces that are conducive for us, the university needs to invest more in ensuring that we have adequate resources within residences so that we can enjoy residence life.’ (P2, female, student, 18–25 years old)

‘[*O*]ur staff and students need to be educated about disabilities and I am happy that as a university we are making progress regarding that which shows that we are serious about issues faced by students with disabilities.’ (P4, female, student affairs practitioner, 41–45 years old)

Participants emphasised the importance of raising awareness about various disabilities, especially among university staff and peers, to promote understanding, inclusivity and better engagement. They also identified the need for adequate resources, particularly in residences, to create environments that support both academic and social participation for students with disabilities (Hlengwa & Masuku 2022). Furthermore, participants suggested that students with disabilities should be encouraged and given opportunities to participate in student governance positions, ensuring their voices are heard and represented:

‘Some do not want to participate in student leadership engagements because of their conditions. As much as we can see potential in them we cannot force them to take part in these activities. Having someone in the Student Representative Council with disabilities could be a big change for us as a university.’ (P7, female, student affairs practitioner, 51–55 years old)

‘… [*S*]tudents with disabilities can be the ones who drive change within our institution and their voice is much more important, especially on issues related to disability. What they need is support from student affairs practitioners.’ (P2, female, practitioner, 41–45 years old)

Empowering students with disabilities to take on leadership roles, like in the Student Representative Council, is seen as a key opportunity to drive institutional change and amplify their voices.

## Discussion

This study’s findings are framed within the socio-ecological framework, which helps understand the multiple factors influencing the experiences of students with disabilities at university. This framework examines the interplay between individual, interpersonal, institutional, community and societal levels, shedding light on the psychosocial, academic and social challenges these students face (Hlengwa & Masuku 2022). While higher education presents challenges for all students, those with disabilities often encounter more pronounced difficulties, balancing academic demands with their unique needs. Scholars such as Salimi et al. (2025) and Solís-García et al. (2025) stress the importance of creating inclusive environments in higher education for students with disabilities. However, factors such as limited access, participation and support continue to hinder their progress compared to peers without disability. This study aimed to explore the perspectives of both students with disabilities and student affairs practitioners on these psychosocial challenges.

### Individual level

In this study, mental health and social challenges were highlighted as the most significant matters that students with disabilities must face. Koenig, McLean and Bishop (2024) stress that people with disabilities have greater psychological distress because of their vulnerability. Several studies have been conducted to compare the mental health needs of students with disabilities to those without disabilities, indicating a significantly higher prevalence of depression, anxiety, non-suicidal self-injury and suicidal risk (Solís García, Real Castelao & Barreiro-Collazo 2024). The findings of this study revealed that these challenges have a detrimental impact on the overall well-being of these students (Hlengwa & Masuku 2022). Disability on its own has a negative effect on an individual, thus, Namkung and Carr (2020), reiterated that students with disabilities face unique challenges that can negatively impact their well-being. It also appeared from the study that poverty is a concerning issue, as some students do not have access to the necessary resources to meet their needs. People with disabilities are the most socially and economically marginalised individuals (Grue 2024). Participants further highlighted that receiving no support from their peers may cause mental health challenges because students with disabilities struggle to relate to others. Charles, Davie and Farai (2024), emphasise that students with disabilities need constant support to manage stress and to cope with mental health challenges and traumatic experiences they often must face. The findings of the study also highlighted that difficulties for students with disabilities in forming relationships can stem from low self-esteem. The finding of this study resonates with the findings of Mamas et al. (2020) that students with disabilities find it difficult to develop relationships with their peers, suggesting that they have fewer friends. While it was suggested that students with disabilities are unable to form relationships with others, it was revealed that the contributing factor is the lack of self-esteem. Self-esteem is an important individual factor because it influences personality and human health and one factor that impacts self-esteem is social support (Lestari & Fajar 2020). The psychosocial challenges that students with disabilities face also contribute to their lack of academic progress, including active participation in extracurricular activities. Mntambo et al. (2024) stressed that students with disabilities are at risk of failing to complete their studies or failing to complete their university degrees in time. This is because of the difficulty in adjusting to the new environment and its expectations, including the challenges they must face in the university and academically. Thus, it is recommended that factors, contributing to the lack of academic progress of students with disabilities, must be identified and given necessary support (Chiu et al. 2019). The study also highlights that students with disabilities lack active participation in extracurricular activities. The importance of students with disabilities’ participation in extracurricular activities cannot be overemphasised. These activities are regarded as social and recreational activities that improve their well-being (Tugli 2015). At the individual level, students with disabilities face challenges such as low self-confidence, mental health issues and the strain of navigating inaccessible infrastructure. The lack of adequate support negatively affects their academic performance and well-being. Providing individualised support, such as counselling and tailored academic interventions, is crucial to addressing these challenges.

### Interpersonal level

The study found that students with disabilities face stigma and discrimination from their peers, who often view their disabilities as a marker of difference (Hlengwa & Masuku 2022). This perception contributes to the exclusion and marginalisation of students with disabilities. According to Dollinger et al. (2024), stigma is a negative belief towards a group of people, which often leads to being threatened by others. This resonates well with the findings of this study as participants confirmed that sometimes students with disabilities are treated inappropriately because of the beliefs people have about disability. It is argued that such experiences involve harmful effects on the well-being of people with disabilities and socio-educational inclusion (Gallego-Ortega & Rodríguez-Fuentes 2021). Furthermore, the discrimination of students with disabilities shows that higher education still poses problems in recognising their rights to equality and for them to be seen. Girli et al. (2016) stress that discrimination prevents a group of people from enjoying their fundamental rights and freedom because of their language, religion, gender and physical differences, which in this case is what students with disabilities are experiencing. The study found that students with disabilities felt they were mistreated because of their condition and often overlooked because others assumed they lacked capability. This calls for universities to strengthen their efforts in achieving inclusive education for all which will ensure that all students are engaged including those with disabilities (Hayes & Bulat 2017). The study also found that the stigmatisation and discrimination of students with disabilities contribute to a lack of confidence and lead to feelings that their emotions and needs are not valued. Thus, student affairs practitioners need to make sure that students with disabilities feel a sense of belonging in the university to eliminate the feeling of not being valued, including their stigmatisation and discrimination. Although Barnes, Kelly and Mulrooney (2021) suggest that having a sense of belonging is a complex and imperative matter that relates to student attainment, it is more challenging for students with disabilities. However, that should not hinder student affairs practitioners’ efforts to address barriers to promoting diversity and inclusion of students with disabilities, especially barriers related to stigma and discrimination (Ramaahlo 2021). The study highlights challenges at the interpersonal level, such as stigma, discrimination and social isolation, which hinder students with disabilities from forming meaningful relationships and engaging in activities. Peers’ lack of understanding contributes to exclusion, underscoring the need for awareness campaigns and peer-support programmes to promote inclusivity and understanding among students.

### Institutional level

Our findings indicate that poor infrastructure and a lack of resources are among the most significant obstacles for students with disabilities on campus (Hlengwa & Masuku 2022). Some lecture venues are inaccessible to these students, leading to missed classes. According to Abrahams (2024), the places where students spend most of their time, such as lecture halls, are often inaccessible to those with disabilities. This finding supports the idea that inadequate infrastructure in the university significantly impacts the university experience for students with disabilities. Achieving inclusive education requires creating a learning environment that is accessible to all students, regardless of their physical, mental or social abilities, or any special educational needs (Messiou 2017). Gow, Mostert and Dreyer (2020) suggest that universities should provide appropriate support to students with disabilities. Some students feel they do not receive adequate support from the university, while student affairs practitioners note that available resources limit the effectiveness of the support provided. The university is working to address these challenges, with initiatives such as establishing disability units that offer specialised services to improve access and integration for students with disabilities. For many of these students, the unit serves as their first point of contact and provides essential support for accessing university life (Mbuvha 2019). Many students with disabilities still feel marginalised because of poor infrastructure and insufficient resources at the university. Inaccessible lecture venues, inadequate accommodations and limited support systems hinder their participation. While progress has been made, stronger institutional commitment is needed, with investments in infrastructure, resources and staff training to create a more inclusive environment (Hlengwa & Masuku 2022).

### Community level

The participants in the study also suggested that students with disabilities should be encouraged and allowed to participate in student governance positions to ensure that their voices are heard and seen. The findings of the study emphasised that students with disabilities have the right to participate in leadership roles, just like their peers without disabilities. Students with disabilities have a right to participate and lead in cocurricular activities, just like students without disability (Tan & Adams 2023). The university community lacks active involvement of students with disabilities in leadership roles and extracurricular activities, isolating them and limiting their impact on institutional change. Encouraging their participation in decision-making bodies, such as the Student Representative Council, can empower them to advocate for their needs and promote a sense of belonging.

### Societal level

Kibret et al. (2025) state that students with disabilities in higher education face many challenges. Students with disabilities suffer a lack of consistent provision for their needs (Ristad et al. 2024). Hence, participants recommended potential intervention and prevention strategies to address the psychosocial challenges of students with disabilities. The last theme that emerged from the study is awareness of disability and the provision of adequate resources. Universities should listen to the concerns of students with disabilities and involve them in curricular and environmental planning (Bartolo et al. 2025; Hlengwa & Masuku 2022). This would demonstrate that the university is working towards inclusivity and diversity. Student affairs practitioners should implement these strategies as they work with students with disabilities. Padden and Ellis (2015) emphasised that university staff must be empowered with knowledge and resources to support students with disabilities effectively. The study’s findings indicate a need for the university to invest more in raising awareness about disabilities to educate the community. Awareness of disability must be made through campaigns and relevant programmes to educate students and staff on disabilities. The creation of awareness of disabilities would play a significant role in eliminating barriers for students with disabilities to participate in university programmes (Moriña & Orozco 2021). Participants also indicated that students with disabilities should be provided with adequate resources to provide for their needs. According to Banks (2022), universities have offices and centres that are specifically designed to provide resources to students with disabilities. Thus, participants also stressed that it is important to provide adequate resources, particularly in residences, to enhance the living experience for these students (Hlengwa & Masuku 2022). The Disability Service offices should play an important role in ensuring that students with disabilities receive direct and indirect support to enhance their well-being and academic excellence (Martins et al. 2016). Societal stereotypes and cultural perceptions about disability contribute to stigma and discrimination faced by students with disabilities. These attitudes are reflected in universities, emphasising the need for cultural shifts. Public campaigns and partnerships with external organisations can challenge ableism and foster a more inclusive society, influencing institutional practices.

### Limitations of the study

The study aimed to explore the perspectives of student affairs practitioners and students with disabilities regarding the psychosocial challenges faced by students with disabilities. While involving both groups strengthened the findings, the study has limitations. The representation of student affairs practitioners exceeded that of students with disabilities, and the racial demographics were skewed, with a majority of black participants and only one white participant. In addition, the findings are specific to the Durban University of Technology in KwaZulu-Natal and cannot be generalised to other institutions of higher learning.

### Recommendations and future research

The recommendations are as follows:

- This study recommends that policies, which speak to disability, must be revised and these policies must highlight inclusivity and diversity, especially around the stigmatisation and discrimination of students with disabilities.
- The study recommends that the university must implement programmes and collaborate with students with disabilities to raise awareness on disabilities.
- Student affairs practitioners must be trained in disabilities to ensure that they are well informed about the challenges of disabilities and how to work effectively with students with disabilities.
- The study also recommends that the university, through student affairs practitioners, must ensure that students with disabilities are provided with all the necessary resources and are given adequate support.
- The university must also ensure that it invests in the infrastructure development that would be suitable to meet the needs of students with disabilities.

The researchers suggest that future studies should focus on the role of student affairs in supporting students with disabilities, specifically addressing their psychosocial challenges. This research would help student affairs practitioners better understand their roles and the interventions needed to assist students with disabilities in overcoming these challenges.

## Conclusion

This study examined the psychosocial challenges faced by students with disabilities, drawing insights from both student affairs practitioners and the students themselves. Four key themes emerged: inadequate infrastructure and resources, stigma and discrimination, mental health and social issues, and the need for disability awareness and support. Guided by the socio-ecological framework, the study emphasised how environmental factors – particularly on campus and in residences – significantly impact the well-being of students with disabilities. The framework’s view that individuals are shaped by their surrounding systems aligns with the findings, confirming that these challenges stem largely from students’ environments and hinder their overall development.

The study found that poor infrastructure and a lack of resources negatively impact students with disabilities. The university infrastructure is not conducive for them as they struggle to move freely on campus, especially when they are supposed to attend lectures or move from campus to campus. The study also highlighted that adequate resources should be made available for students with disabilities and these resources are important to them to cope with and experience university life positively. Furthermore, students with disabilities experience stigmatisation and discrimination from their peers because of their disability. The findings reveal that disability is still perceived as unusual by some, highlighting the need for universities to support students with disabilities. These students face mental health and social challenges, contributing to a sense of vulnerability and negatively affecting their academic performance and relationships. The study emphasises the importance of creating an inclusive environment to foster belonging and prevent mistreatment. Participants proposed interventions such as increasing disability awareness and offering sufficient resources to address these issues. Empowering students with disabilities through targeted support can help overcome psychosocial barriers and promote their overall well-being and success in academic settings. The study recommends that the university implements disability awareness programmes and provides sufficient resources to enhance the experiences of students with disabilities.
